# Genomic profiles of *Pyricularia oryzae* in Sub-Saharan Africa: exploring population differences and their evolutionary implications in the region

**DOI:** 10.3389/fpls.2025.1650532

**Published:** 2025-10-06

**Authors:** Geoffrey Onaga, Worrawit Suktrakul, Margaret Wanjiku, Ian Lorenzo Quibod, Jean-Baka Domelevo Entfellner, Joseph Bigirimana, Georges Habarugira, Rosemary Murori, Godfrey Asea, Hamidatou Euridice Tella, Gbenakpon Aubin G. Y. Amagnide, Negussie Zenna, Chatchawan Jantasuriyarat, Abdelbagi M. Ismail, Ricardo Oliva

**Affiliations:** ^1^ Africa Rice Center (AfricaRice), Mbe Research Station, Bouake, Côte d’Ivoire; ^2^ Department of Genetics, Faculty of Science, Kasetsart University, Bangkok, Thailand; ^3^ Biosciences Eastern and Central Africa-International Livestock Research Institute (BecA-ILRI) Hub, Nairobi, Kenya; ^4^ Rice Breeding Platform, International Rice Research Institute, Metro Manila, Philippines; ^5^ Rice Breeding Platform, International Rice Research Institute, Bujumbura, Burundi; ^6^ Rice Breeding Platform, International Rice Research Institute, Nairobi, Kenya; ^7^ National Crop Resources Research Institute (NaCRRI), National Agricultural Research Organization, Kampala, Uganda; ^8^ Biodiversity for Food & Agriculture, International Centre for Tropical Agriculture (CIAT), Alliance Biodiversity and CIAT, Americas Hub, Cali, Colombia

**Keywords:** blast effectors, evolution, genetic origin, single nucleotide polymorphism, rice

## Abstract

**Background:**

Rice blast, caused by *Pyricularia oryzae* (teleomorph: Magnaporthe oryzae), is one of the most economically damaging diseases affecting rice worldwide. While the evolutionary origins and genetic structures of Asian and European P. oryzae populations are relatively well characterized, African isolates remain underexplored. This knowledge gap impedes the development of informed management strategies for rice blast in the region. The present study was conducted to characterize the genetic origins, population structure, admixture, demographic history, and effector gene diversity of P. oryzae isolates in SSA, and to elucidate their evolutionary trajectories and implications for disease management.

**Methods:**

A total of 180 genome sequences (45 from SSA, 135 from other regions) were analyzed using population genomic approaches. Phylogeographic reconstructions, demographic modeling, and genome-wide association studies were performed to trace migration events, quantify genetic diversity, and identify candidate adaptation genes. Effector gene repertoires were also examined for diversity and selection signatures.

**Results:**

Our findings provide new dates for the divergence of SSA populations from Asian populations. The introduction of P. oryzae into Africa occurred mainly from China in the late 19th century, initially in West Africa (WA; Mali and Burkina Faso), and subsequently in Uganda and Madagascar during the early 20th century, before extending to the wider African region, with subsequent repeated introductions. Tajima’s D and demographic modeling suggested complex population dynamics shaped by migration and asymmetric founder events, highlighting considerably shared genetic ancestry between Asia and East Africa (EA), in contrast with that between Asia and WA. Genome-wide association analysis identified a specific set of single nucleotide polymorphism markers, along with several candidate genes linked to adaptation. Effector analysis revealed that SSA isolates harbor fewer effectors and exhibit lower genetic diversity than Asian populations, with some effectors under positive selection, particularly in WA.

**Discussion:**

P. oryzae populations in SSA are shaped by historical introductions, founder events, and region-specific adaptation processes. While WA populations have diverged significantly from their Asian ancestors, gene flow within SSA connects regional populations, and effector gene diversity reflects both conserved virulence strategies and adaptation to local hosts. Overall, this study improves the existing knowledge on P. oryzae populations in SSA and underscore the need for integrated management strategies that consider both historical and contemporary pathogen dynamics in Africa.

## 1 Introduction

Routine genomic surveillance of plant pathogens across diverse geographic regions, along with equitable data sharing, is essential for effective plant disease management and timely outbreak response. However, most countries in Sub-Saharan Africa (SSA) remain markedly underrepresented in large-scale pathogen genomic studies, resulting in limited regional capacity for pathogen monitoring and an increased vulnerability to emerging disease threats (Mutiga et al., 2021; Conde et al., 2025). The dynamics of plant diseases are shaped by intricate interactions between the host, environment, and pathogen communities, with local adaptation and evolutionary change often influencing disease emergence and severity (Stukenbrock and McDonald, 2008; Mundt, 2014). Given that the evolution of pathogens is frequently driven by localized ecological and agricultural factors, region-specific genomic studies are critical for validating biological processes and informing tailored disease management strategies.

Rice blast, caused by *Pyricularia oryzae* (syn. *Magnaporthe oryzae*), is among the most destructive and historically significant rice diseases worldwide, having first been reported in China as early as 1637 (Agrios, 2005). The pathogen is now endemic in over 100 countries across Asia, Africa, the Americas, Europe, and the Middle East (CABI, 2021; Pedrozo et al., 2025). Yield losses due to blast range from 10–30% under typical conditions but can reach 40–100% during severe epidemics, not only in rice but also in wheat and turfgrass (Savary et al., 2019; Vanaraj et al., 2013; Coelho et al., 2016; Skamnioti and Gurr, 2009; Uddin et al., 1999). As a polycyclic pathogen, *P. oryzae* can complete multiple infection cycles per season, amplifying its destructive potential, especially in favorable or previously unexposed cropping systems (Ribot et al., 2008). Recent trends in rice production, including reduced water usage, expansion of high-yielding cultivars, and increased fertilizer application, are intensifying selection pressures and increasing pathogen evolution (Mutiga et al., 2021). Analogous trends have been observed in other major crop diseases, such as the emergence and global spread of the highly virulent Ug99 lineage of wheat stem rust, where changes in agricultural practices and host deployment have accelerated evolution and dispersal (Singh et al., 2011). These examples underscore the urgent need for robust, ongoing genomic surveillance of plant pathogens to detect shifts in genetic diversity and inform sustainable crop protection strategies.

The genetic diversity of *P. oryzae* is shaped by mutation, genetic drift, gene flow, sexual and asexual reproduction, and strong selection from host resistance genes (McDonald and Linde, 2002). These evolutionary forces not only drive pathogen adaptation and virulence but also provide the foundation for reconstructing the pathogen’s demographic history, migration routes, and mechanisms underlying virulence evolution. Southeast Asia has been established as both a biodiversity hotspot and the likely center of origin for *P. oryzae*, from which host-specific lineages have radiated during historical host transitions (Saleh et al., 2014; Couch et al., 2005). The rice-infecting lineage is thought to have arisen approximately 1,000 years ago from a recombining grass-infecting ancestor, following a single gain-of-function event for rice pathogenicity (Gladieux et al., 2018). Genomic surveys have subsequently revealed four major genetic groups within the rice-infecting lineage, with distinct global distributions and reproductive strategies: Group 1 is characterized by recombination and sexual reproduction, while Groups 2, 3, and 4 are largely clonal and female-infertile (Gladieux et al., 2018; Thierry et al., 2022).

Dating studies indicate that the major clonal expansions of *P. oryzae* occurred within the last 100–200 years, originating from genetically diverse populations in Southeast Asia (Duan et al., 2024; Latorre et al., 2020). These expansions have shaped the current genetic profiles of Asian populations, resulting in the formation of distinct genetic groups and a marked reduction in effector gene repertoire compared to the ancestral, more diverse populations. Bottleneck effects and founder events during such clonal expansions lead to the loss of rare alleles and a narrowing of both adaptive and neutral genetic variation, a trend reported in other plant pathogens (Wilson et al., 2025; Saleh et al., 2014). Similar processes appear to have influenced *P. oryzae* in regions outside Asia, including SSA, where introductions are inferred to have been relatively recent compared to the pathogen’s much earlier establishment and diversification in Asia (Thierry et al., 2022). However, many African countries remain severely undersampled, and most studies have used low-resolution markers, limiting inferences about population history and finer-scale structure (Kassankogno et al., 2016; Onaga et al., 2015). A recent bibliometric review highlighted that Africa has the lowest research output on rice blast and related rice diseases compared to other continents (Conde et al., 2025). As a result, significant knowledge gaps persist about *P. oryzae* in Africa, compounding the lack of comprehensive data needed for informed disease management.

Historical records first identified rice blast symptoms in SSA in Uganda in 1922 (Small, 1922), with subsequent detections in Ghana (1923), Kenya (1924), Congo (1932), Egypt (1935), and later in Madagascar, Morocco, Senegal (1952), and South Africa (1956) (Asuyama, 1965). More recently, global population studies employing genotyping and lineage analysis, including some genomes from West African countries, have revealed the presence of all four major genetic groups of *P. oryzae*, underscoring the broad and diverse nature of the pathogen population in Africa (Thierry et al., 2022). Previous research using larger collections of SSA isolates identified considerable genetic diversity and evidence of population structure across SSA, with subtle differentiation observed between East and West African populations (Odjo et al., 2021; Kassankogno et al., 2016; Onaga et al., 2015; Mutiga et al., 2017). However, many of these earlier studies relied on low-resolution genetic markers, such as simple sequence repeats (SSRs) or partial genome data, which only provide a limited view of genetic diversity and are insufficient for reconstructing the demographic and migration history of the pathogen. To overcome these limitations, high-resolution whole-genome studies are needed to clarify the relationships between African and Asian populations, establish the timing and routes of introduction, and guide effective disease control.

Rice blast control in Africa, as elsewhere, relies primarily on the deployment of resistant rice varieties. However, the durability of resistance is frequently compromised by the rapid emergence of new, resistance-breaking strains, a direct consequence of ongoing pathogen evolution driven by mutation, gene flow, and selection imposed by host resistance genes (McDonald and Linde, 2002). The capacity for *P. oryzae* to gain, lose, or rearrange genes, particularly those encoding effectors, enables it to evade host immunity and adapt quickly to new resistant cultivars (Yoshida et al., 2016; Chiapello et al., 2015). This dynamic nature of effector gene evolution, through gene gain/loss, diversifying selection, and genetic drift, means that previously effective resistance genes can rapidly become obsolete as the pathogen adapts (Yoshida et al., 2016). Given these challenges, regular monitoring of *P. oryzae* populations, particularly with a focus on effector diversity and evolution, is crucial to inform breeding programs and ensure long-term disease management (Dong et al., 2015; Latorre et al., 2020). Unfortunately, most effector diversity studies have concentrated on Asian populations, with African strains remaining severely underrepresented in global analyses. This data gap constrains our ability to track virulence evolution, predict resistance breakdown, or tailor management strategies to the specific evolutionary dynamics present in Africa.

In this study, whole-genome sequences from 45 P*. oryzae* isolates collected across 14 African countries were analyzed alongside 135 genomes from other global regions to explore population variation in SSA. Unlike earlier studies, different evolutionary scenarios were tested to provide a more comprehensive regional perspective on *P. oryzae* and to improve understanding of its introduction and spread within SSA. In addition, effector polymorphisms in SSA isolates were compared at both continental and regional levels. Although SSA isolates clustered with Asian strains, varying degrees of divergence were exhibited, alongside distinct patterns of effector gene presence/absence and evidence of selection. These results improve our understanding of *P. oryzae* populations in SSA and highlight the need for continous monitoring of genetic variations that could cause disease epidemics.

## 2 Materials and methods

### 2.1 Collection of *P. oryzae* isolates and DNA extraction

In this study, 45 isolates were collected from 14 African countries, including Burundi (4), Kenya (2), Rwanda (3), Tanzania (9), Uganda (5), Benin (4), Burkina Faso (4), Ghana (2), Mali (2), Nigeria (4), Togo (3), Madagascar (1), Morocco (1), and Côte d’Ivoire (2). Additional isolates were obtained from Asia (4) and Latin America (3). The metadata for the isolates, including those downloaded from public INSDC databases, are provided in **Supplementary Table S1**. Infected leaves were collected from rice fields, dried, and stored on filter paper at 4 °C before isolation. Leaves with single lesions were placed on glass rods in Petri dishes containing wet filter paper and incubated at 25–26 °C until sporulation was achieved. Sporulating lesions were examined under a stereomicroscope, and conidia were aseptically transferred with a transfer needle to prune agar (PA) medium (composed of three pieces of prunes, 1 g yeast extract, 21 g Gulaman bar, 5 g alpha-lactose monohydrate, and 1 L distilled H_2_O) (Meng et al., 2020). Spores were then harvested in distilled water, and individual germinating conidia were aseptically transferred and subcultured in PA medium. After seven days, agar disks cut from PA were subcultured on Malt Extract Agar. Fungal growth and DNA extraction were performed as previously described (Mutiga et al., 2017). The quality of DNA was checked using a NanoDrop 1000 instrument (Thermo Fisher Scientific) and agarose gel electrophoresis.

### 2.2 Generation of genomic datasets

The sequence datasets used in this study were obtained from *P. oryzae* isolates collected in the field or downloaded from the public INSDC databases. For the isolates sampled from the field, DNA samples were submitted and library construction was performed by the Beijing Genomic Institute (Shenzhen, China) using Illumina paired-end reads with an insert size of 350 bp. Sequencing was performed on Illumina HiSeq4000, with sequencing parameters set to an average coverage of 50x to 70x. Sequencing quality was confirmed using the fastqc algorithm, and the data were trimmed by removing low-quality sequences and adapter sequences with Trimmomatic 0.36 (Bolger et al., 2014). The whole-genome sequence of *P. oryzae* 70–15 strain, reference assembly MG8 with accession number GCA_000002495 (Dean et al., 2005), was used as the reference template for mapping using BWA–mem 0.7.17 (Li and Durbin, 2009), under default parameters. Mapped reads were sorted with Samtools 1.3.1 (Li et al., 2009). Duplicate reads were removed using the *MarkDuplicates* command, and all reads in a file were assigned to a single new read-group using the *AddOrReplaceReadGroups* command with Picard 2.7 (http://broadinstitute.github.io/picard). Single nucleotide polymorphisms (SNPs) for each strain were identified using the *HaplotypeCaller* command implemented in the Genome Analyses Toolkit 4 (GATK4.1.6.3) (McKenna et al., 2010). Subsequently, the *GenotypeGVCFs* command in GATK was simultaneously applied to the genotype polymorphic sequence variants for all strains. Hard-filtering was performed for raw SNP calls using the *SelectVariants* and *VariantFiltration* functions in GATK (De Summa et al., 2017). The following parameters were used for variant calling: QD < 5.0, QUAL < 5000.0, MQ < 20.0, ReadPosRankSum < -4.0, ReadPosRankSum > 4.0, MQRankSum < -2.0, MQRankSum > 2.0, BaseQRankSum < -2.0, BaseQRankSum > 2.0. The final SNP dataset was further filtered to only include biallelic variations. *P. oryzae* isolates with a mapping rate of less than 80% to the reference strain were excluded from the population genetic analyses; however, all reads were used for effector mapping. Genomic datasets for 131 P*. oryzae* isolates from a global population (Gladieux et al., 2018b; Zhong et al., 2017) were downloaded from the Sequence Read Archive (http://www.ncbi.nlm.nih.gov/sra). A summary of the sequencing yield and dataset coverage is presented in **Supplementary Table S2**.

### 2.3 Phylogenetic and phylogeogoraphy analysis

Phylogenetic relationships and phylogeography were investigated using maximum likelihood (ML) and Bayesian inferences with RAxML 8.2.9 (Stamatakis, 2014) and BEAST v2.6.3 (Bouckaert et al., 2019), respectively. For the ML tree, the statistical confidence for each node was set to 1000 bootstrap runs, and the general time-reversible model of nucleotide substitution with a gamma model of rate heterogeneity was used. The phylogenetic tree was visualized using the *ggtree* R package (Yu et al., 2017), and visualization was improved using iTOL v5 (Letunic and Bork, 2021). For phylogeographic analysis, the most appropriate substitution model, General Time-Reversible (GTR+G+I), was identified by the *jModelTest v2.1.10* program (Posada, 2008). Bayesian maximum-clade-credibility (MCC) phylogenetic trees were constructed as described by Drummond and Rambaut (2007). To infer the geographic origins of *P. oryzae* strains, location and genetic group traits were analyzed using a discrete trait diffusion model. To estimate the transition rates between different locations, a Bayesian stochastic search variable selection (BSSVS) with a symmetrical discrete trait substitution model (strict clock assumption) was used. The Markov chain Monte Carlo (MCMC) was run under an Extended Bayesian Skyline model for over 100 million generations, with sampling conducted every 1,000 states. For the major parameters, an effective sample size (ESS > 200) was observed. The ESS values were evaluated using TRACER v1.8.4, and a cutoff value of 200 was applied to retain the concluding simulations. TreeAnnotator v1.8.4 (after removal of 10% burn-in) was used to generate the MCC tree. Ultimately, the resulting MCC tree was visualized using FigTree v1.4.3 (http://tree.bio.ed.ac.uk/software/figtree/). A posterior probability threshold (pp) higher than 0.95 was used to assess the tree nodes with SPREAD3 (Bielejec et al., 2011). SPREAD3 was also used to calculate the Bayes factors (BF) for pairwise diffusion rates between sites. Bayes factors greater than 3 were considered statistically significant. The simulations of 300 million iterations produced better results than the 100 million simulations. Phylogenetic analysis results were projected onto a map using Google Earth™ to enable visualization and understanding of the relationship between pathogen genomes and geographical locations. This was accomplished by creating a Keyhole Markup Language (KML) file, and importing and converting in shapefiles in QGIS. which allowed visualization of *P. oryzae* spread and genetic relationships on a map.

To further support the phylogenomic relationships and probe deeper into the genetic partitioning of the datasets, several clustering approaches were used. The whole-genome neighbor-net network analysis was employed using the neighbor-net method implemented in SplitsTree 4.16.1 (Huson and Bryant, 2006). Assignment tests were conducted using principal component analysis (PCA; employing the genlight object and the glPCA function from the adegenet package in R followed by STRUCTURE v2.3.4 (Pritchard et al., 2000) and discriminant analyses of principal components (dAPC) also using the “adegenet” v1.31 R package (Jombart et al., 2008). STRUCTURE was run for K values ranging from 1 to 10, with each K value evaluated using 20,000 Monte Carlo Markov Chain (MCMC) iterations, following a burn-in period of 10,000 iterations, across ten replicate runs. As dAPC requires prior population information, inferred population assignments from STRUCTURE and phylogenetic relationships observed in the PCA were used. A dAPC was performed using the “dapc” function, whereas the number of clusters (K) with the lowest Bayesian information criterion (BIC) was determined using the “find.clusters” function within the “adegenet” package. The R package factoextra was used to validate the optimal number of clusters in a silhouette plot (Kassambara, 2017).

### 2.4 Linkage disequilibrium, GWAS, Admixture, and genetic diversity analysis

Following the identification of the optimal number of clusters, isolates were reclassified into four populations according to their region of origin: EA (EA), WA (WA), Asia (A), and the rest of the world (ROW), and their ancestry was assessed. SNPs were filtered from the original VCF file using three different cutoffs of presence for all samples (1%, 5%, and 10%), and new VCF files were then generated for each cutoff. To investigate the relatedness between samples in the filtered SNP VCF files using the TASSEL package (version 5.2.86, Bradbury et al., 2007), four principal components were extracted, capturing the most significant variations in the genetic data. The PCA results, specifically the scores of the samples on the four principal components, were exported as a NumPy matrix, which was then imported into a Python environment, where visualization was carried out using the NumPy and Matplotlib libraries. Variance of up to 60% was represented by PCs 1 and 2, and four genetic groups were confirmed based on genetic relatedness. Linkage disequilibrium (LD) between pairs of SNPs from isolates across the four regions was assessed by calculating the mean LD within 10 Kb end-to-end sliding windows genome-wide, with each window centered on a given SNP. SNP pairs were grouped into bins based on physical distance (in base pairs) or chromosomal proximity. SNPs with low minor allele frequencies (MAF ≥ 0.1) were excluded prior to binning and subsequent analysis in conjunction with the PCA-derived genetic groups. For each bin, the average r^2^ (half decay distance) was determined by summing the r^2^ values for each pair of SNPs within the bin and dividing by the total number of SNP pairs present in that bin. SNPs were pooled across all chromosomes within each genome to determine the overall average LD on a genome-wide scale. LD between SNPs was assessed by plotting their average r^2^ values, with minimal LD expected at r^2^ values of approximately 0.2. The binned SNPs and PCA-derived pathogen genetic groups were subsequently used as input for genome-wide association studies (GWAS) in TASSEL to identify associations. The results of the GWAS were then analyzed to identify a list of SNPs significantly associated with pathogen groups, with LD decay taken into account to refine the identification of truly associated SNPs. These SNPs were further analyzed to identify a list of genes containing SNP variants, and a VCF file with all required annotations was generated.

Admixture analysis was performed using the ADMIXTOOLS package (version 7.0.2; Patterson et al., 2012) within R environment (R Core Team, 2023). f2 statistics were calculated by measuring the squared allele-frequency distance between pairs of genetic groups subdivided into regions. As the f2-distances are squared Euclidean, pairwise f2(Xi, Xj) values between all populations were calculated and assembled into a matrix, resulting in a three-dimensional (3D) array containing 24 distance matrices. From these matrices, 24 trees were generated using the PHYLIP package (version 3.698; Felsenstein, 1989), employing the neighbor-joining method implemented in the neighbor program. To account for variability across the matrices, a consensus tree was constructed from the 24 individual trees using the consense program in PHYLIP, following standard consensus-building procedures (Felsenstein, 1989). To further investigate population structure and admixture, we performed the 3-Population Test (outgroup f3-statistics), as implemented in ADMIXTOOLS. This test measures the amount of shared genetic drift between two test populations relative to an outgroup and is robust for detecting admixture events. For these analyses, we used US 71, a Setaria-infecting *P. oryzae* strain, as the outgroup, consistent with previous studies (Gladieux et al., 2018a). The resulting f3-statistics were used to generate a pairwise genetic similarity matrix, which was then subjected to complete linkage hierarchical clustering using the *hclust* function in R. Cluster tree was visualized with the *ggtree* package (Yu et al., 2017), and additional graph depicting genetic distances between regions was created using the *ggplot2* package (Wickham, 2016).

The VCFTOOLS software, with the “*-haploid*” flag (downloaded from https://github.com/jydu/vcftools), was used to calculate F_st_ statistic, diversity index (Pi), and Tajima’s D (Tajima, 1989). Variations within the different genetic groups at the 10% SNP presence cutoff were calculated, and boxplots for Pi index, F_st_ statistic, and Tajima’s D were plotted. For F_st_ outlier analysis, F_st_ values were estimated for each 10 kb-sized window, resulting in the generation of an F_st_ value for each individual SNP by comparing regional populations. Genes from each comparison (EA-WA, A-EA, A-WA, and A-ROW) were extracted to predict their biological role in pathogen adaptation.

### 2.5 Population genetic analyses of demography

The demographic patterns of *P. oryzae* populations were investigated based on the folded site frequency spectrum (SFS) using the diffusion approximation framework implemented in ∂a∂i (Gutenkunst et al., 2009). This model assumes that an ancestral population of size X gives rise to two populations of size X1 and X2, respectively, at a time of split Ts, after which several migration scenarios are contrasted (Momigliano et al., 2021). The SFS, representing the distribution of allele frequencies across SNPs, was used to infer the demographic history of populations. The observed SFS was compared to expected SFS values generated under different demographic models, and the likelihood of each model, along with its parameter values, was estimated using maximum likelihood methods. Each geographic region (EA, WA, and Asia) was treated as a subpopulation. The ROW subregion was excluded due to insufficient sample size and potential population substructure, which could bias the SFS and downstream inferences (Excoffier et al., 2013). Multiple projection sizes were evaluated for each population, and the projection that retained the largest number of SNPs without excessive missing data was selected for further analyses. Demographic models were fitted to the observed SFS for each population, including, Expansion (two-epoch), Growth, Bottle-growth and bottleneck-expansion (three-epoch function) (Gutenkunst et al., 2009). For each model, initial parameter values were randomly drawn from uniform distributions within biologically plausible bounds, following Gutenkunst et al. (2009). The model with the highest log-likelihood was selected as the best-fit for each region. To investigate historical relationships between population pairs, we constructed two-dimensional (joint) SFS using pairs of regions: Asia-EA, Asia-WA, and EA-WA. We fit two-population demographic models, including split-migration and isolation-with-migration scenarios, to each pairwise SFS (Gutenkunst et al., 2009; Excoffier et al., 2013). Each model was optimized iteratively, and the best-fit parameters were determined based on maximum likelihood criteria. For each fitted model, we compared the log-likelihood values to assess relative model fit. Model adequacy was visually evaluated by plotting the observed vs. expected SFS, as well as the distribution of residuals. These plots allowed us to assess the extent to which the best-fit model captured the main features of the data.

### 2.6 Effector mapping, distribution, and diversity

To map candidate effectors in SSA *P. oryzae* genomes, previously described methods and resources (Latorre et al., 2020) were followed with modifications. Protein-coding genes, both virulent and avirulent, from *P. oryzae* isolates infecting rice, wheat, oat, millet, and wild grasses were used to generate reference effector sequences (Petit-Houdenot et al., 2020). Genome reads from 180 P*. oryzae* isolates were mapped to the effector reference using bwa-mem 0.7.17 (Li and Durbin, 2009). Mean gene coverage for each isolate was calculated with samtools coverage (v1.10), using a minimum read depth of 3x. The total number of mapped reads per gene was divided by gene length in the reference (Li et al., 2009), and an 80% coverage threshold was set to define effector presence. A binary presence/absence matrix was generated. Only informative effector genes displaying presence/absence polymorphisms were retained for clustering. Hierarchical clustering was performed using the hclust function with complete linkage, and distance matrices were computed in R with the ade4 package (Dray and Dufour, 2007), employing the dist.binary function and Jaccard index. Principal component and effector loading analyses were conducted as described previously (Latorre et al., 2020). Effector bam files were converted into fastq format using samtools 1.10 and bcftools 1.10 (Li et al., 2009) for variant calling and diversity estimation. Sequences with zero presence, ambiguous nucleotides (“N”), unknown bases, or heterozygous positions were excluded. Effector alignments were generated using MAFFT 7.453.0 (Katoh and Standley, 2013) with the G-INS-i strategy and were manually curated. Effector diversity indices were calculated with the R package pegas (Paradis, 2010), using hap.div for allele diversity and nuc.div for nucleotide diversity (Pi).

Synonymous and nonsynonymous substitution rates, as well as site-specific positive selection, were estimated using YN00 and CODEML from the PAML suite. Orthologous effector genes were identified using BLAST, and sequence alignments were generated for each set. For genes with at least two orthologs, we calculated mean pairwise dN/dS ratios using KaKs_Calculator 2.0 (Wang et al., 2010) with the Yn00 model (Yang et al., 2000). Additionally, site-specific positive selection was evaluated using CODEML (version 4.10.6) from the PAML suite. Likelihood ratio tests (LRTs) were performed, comparing models M1 (neutral) vs. M2 (selection) and M7 (beta) vs. M8 (beta&ω), with statistical significance determined by chi-square tests (P < 0.05). A gene was considered under site-specific positive selection if both LRTs were significant. Preliminary examination of effector sequences revealed >99% identity among samples, and further analysis was performed by repeating CODEML tests with an outgroup, *Magnaporthe poae*, selected for sufficient evolutionary distance and homology. Orthologous effector gene sequences from this outgroup were analyzed individually. The dN/dS ratios were calculated only for proteins with corresponding orthologs in *Magnaporthe poae*, and region-wise comparisons were conducted for each effector gene. Results were visualized using boxplots.

## 3 Results

### 3.1 Phylogenetic and phylogeography analysis

**Figure 1:**
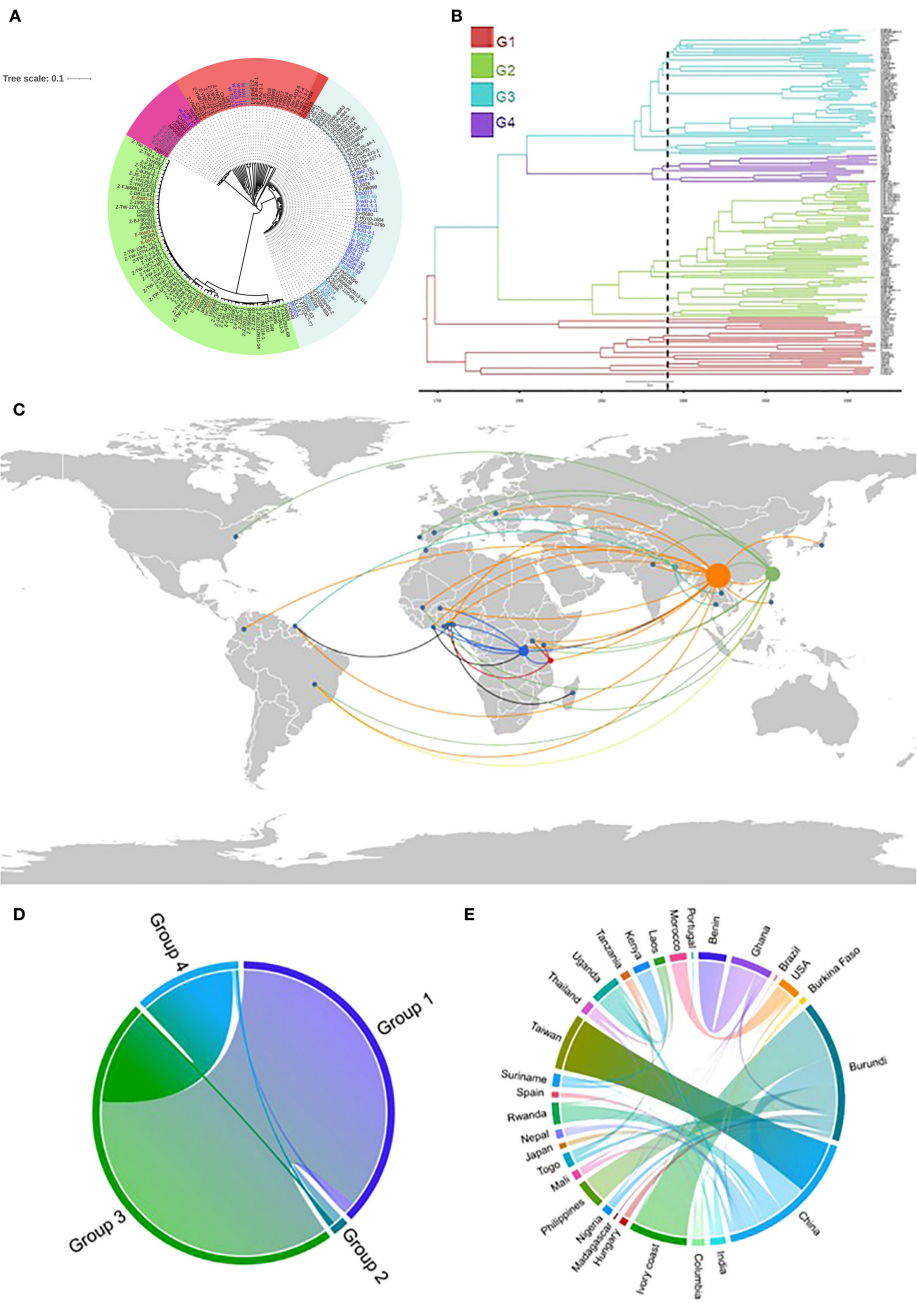
**(A)** A maximum-likelihood phylogenetic tree generated using single nucleotide polymorphism (SNP) alignment of global isolates. Font colors on the tree represent SSA isolates in different clusters (genetic groups) **(B)** Bayesian maximum clade credibility (MCC) tree from BEAST based on 66,744 SNPs among the global population; genetic groups are represented by the different colors. **(C)** A Google Earth map created using Keyhole Markup Language file allowing for visualization of the spread of *Pyricularia oryzae* and genetic relationship between pathogen genomes and geographical locations. **(D)** Cord diagram generated using a Bayesian stochastic search variable selection, with a symmetrical discrete trait substitution model (strict clock assumption) to estimate the transition rates between groups **(D)** and different locations **(E)**.

To characterize the genetic composition of SSA populations of *P. oryzae*, previous datasets (Gladieux et al., 2018a; Zhong et al., 2017) were combined with genome sequences from newly collected isolates in SSA rice-growing regions. In the entire collection, 66,744 SNPs were identified. The phylogenetic signal of SSA strains was analyzed using ML. SSA genomes clustered within three well-defined global genetic groups, as well as within a diverse Group 1 (**Figure 1A**). Most Group 1 isolates were more broadly related to Groups 3 and 4, as indicated by shared branching points, whereas Group 2 appeared to be relatively distant. Group 1 was represented by five SSA isolates (SSA-1): a single isolate from East Africa (Uganda; subclade 1) and four from West Africa (Mali, Togo, Ghana, and Nigeria; subclade 2) that grouped together with Asian isolates predominantly from Yunnan, China (13FM-5-1, 13FM-24-1, CH1019 and CH0999). Most SSA isolates were assigned to Group 3 (SSA-3), which was detected across all SSA regions. Group 4 (SSA-4) included isolates from Tanzania, two from Burkina Faso, and one each from Uganda and Kenya. Group 2 (SSA-2) was restricted to East Africa and comprised mainly isolates from Rwanda and Burundi, along with one isolate each from Uganda and Morocco. The ML tree was compared with a tree reconstructed using the maximum clade credibility (MCC) method in BEAST2. The time-calibrated MCC phylogenetic tree closely resembled the ML tree (**Figure 1B**). The most recent common ancestor (MRCA) of the analyzed samples was estimated to have emerged around 1742 (95% highest probability density (HPD): 1700–1753) (**Supplementary Table S3**). Divergence into clonal groups was dated to approximately 1748, leading to emergence of Group 1 and clonal Group 3. Another divergence, resulting in Group 2, occurred in 1786, followed by the emergence of Group 4 in 1804. The MRCA for SSA samples was traced to 1896 in Burkina Faso, West Africa (95% HPD: 1891–1917), with subsequent emergence in Mali between 1896 and 1900. For East Africa, the MRCA was identified in Uganda in 1910 (95% HPD: 1905–1929), with emergence in Madagascar seven years later (95% HPD: 1900–1918). Through Google Earth visualization, we observed that several SSA isolates were likely originated from China, while others were inferred to have previously circulated in East Asian countries, such as South Korea, before their emergence in SSA (**Figure 1C**). Evidence of a reintroduction event involving China and Burundi in the 1950s was observed, which was subsequently followed by introductions to Ghana and Burkina Faso in the late 1960s. Substantial migration flux between EA and WA was detected in the early 1990s, particularly involving Tanzania, Burundi, and several West African countries.

To verify these geographic transitions, a discrete trait diffusion model was implemented. A considerable representation of Group 1 alleles in Group 3 was observed, whereas Group 2 showed reduced representation across all groups (**Figure 1D**). Most *P. oryzae* isolates in SSA were consistently traced to migration from China and India (**Figure 1E**). In EA, strong connections were found between Uganda and Kenya, with each also maintaining separate links to Tanzania. Within WA, Ghana exhibited a robust connection with Benin. Madagascar displayed limited connectivity with other countries, apart from a distinct link with Benin. Transition rates indicated that Burundi acted as a repository for isolates from various countries, particularly from Côte d’Ivoire, Burkina Faso, Ghana, China, the Philippines, and Nigeria.

### 3.2 Population structure and admixture analysis

**Figure 2:**
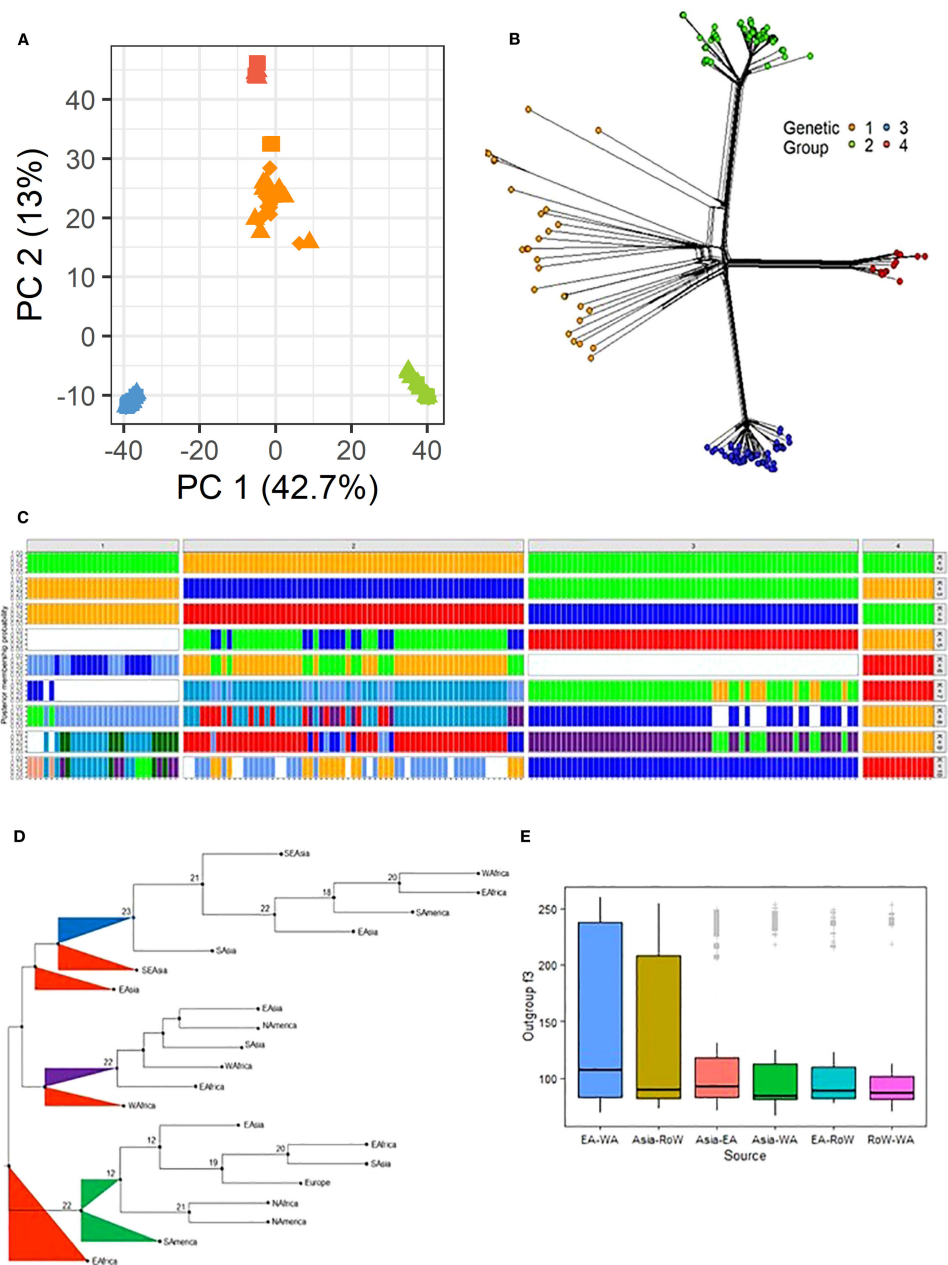
**(A)** A two-dimension principal correspondence analysis showing the distribution of genetic variability in the *Pyricularia oryzae* datasets. **(B)** Phylogenetic network analysis of global *P. oryzae* populations using the neighbor net method showing the four inferred genetic groups. The color denotes the four genetic groups as inferred using the PCA in panel A and confirmed via the clustering analysis in **Supplementary Figures S1B, C**. **(C)** STRUCTURE results showing the membership probability for each *P. oryzae* genome from K = 2 to K = 10 populations. **(D)** A consensus tree generated using f2 statistics between all pairs of groups subdivided into regions using the PHYLIP package. **(E)** A 3-Population test or outgroup f3-statistics implemented in AdmixTools to estimate the genetic similarity between isolates relative to an outgroup; genetic distance between regions are presented in a graphical form.

Population structure and admixture analyses provide insights into the genetic composition of populations, elucidating patterns of relatedness, migration, and the degree of genetic exchange among different groups. In this study, principal component analysis (PCA) clustered the isolates into four groups, consistent with the phylogenetic analysis (**Figure 2A**). The first principal component accounted for the majority of variance (42.7%) and separated Group 1 isolates from those in other groups, while the second principal component explained 13% of the variance and further distinguished the four groups identified in the phylogenetic tree. In PC space, isolates in Group 4 were effectively clustered above those in Group 1. Exceptions were observed for isolates HB-LTH18 from Hubei and CH1016 from Yunnan, which, although grouped within Group 1 in the PCA, appeared closer to Group 3 in the phylogenetic analysis. Similar clustering patterns were revealed by phylogenetic network analysis using the neighbor-Net method, indicating that both approaches captured consistent data structures (**Figure 2B**). Subsequent analysis with STRUCTURE showed that SSA isolates clustered with Asian populations across all groups (**Figure 2C**). As the number of genetic groups (K) increased to 10, a gradual seperation appeared between Asian and SSA populations in each group, particularly in group 1 and 2. Discriminant analysis of principal components (dAPC) supported a model with K = 4 genetic clusters, as determined by both BIC and silhouette plot cross-validation (**Supplementary Figure S1**).

Admixture analysis was performed to determine the regional ancestry of the isolates, using genetic distances calculated from f2 statistics between all pairs of genetic groups (G1–4) subdivided by region. A total of 24 trees were generated from the f2 statistics-derived distances, and a consensus tree was constructed (**Figure 2D**). The foundational population for Group 3 was identified as Group 1 from Southeast Asia (SEA). Before the formation of the final SSA-3 (EA and WA) cluster, two distinct intermediate clusters from South Asia and East Asia were observed. The origins of SSA Group 4 (SSA-4) were traced to the lineage of Group 1 in China, which appeared to have originated from a broader Group 1 population. WA Group 1 was found to have diverged before the emergence of Group 4 strains that are were detected in other regions, including South America, North America, East Asia, Europe, WA and EA. EA. The ancestral Group 1, from which EA Group 1 diverged, was positioned at the base of the branch that was linked with Group 2. A weak connection was detected between EA Group 1 and other SSA groups, even within Uganda. Similarly, Group 2, which was found to have emerged from the same ancestral population as EA Group 1, was observed to be distant from Groups 3 and 4.

Further analysis using f3-statistics was conducted to evaluate the genetic similarity between two populations, as inferred with a distant outgroup. Positive f3 values (Z-score > 2) were observed for all genetic groups (**Supplementary Table S4**). At the regional level, higher f3 statistics were found between EA and WA and between Asia and ROW (**Figure 2E**).

### 3.3 Linkage disequilibrium and GWAS analysis

To investigate the extent of clonal reproduction in our collection, linkage disequilibrium (LD) decay was evaluated by calculating the squared correlation coefficient (r²) between pairs of SNPs using the TASSEL interface (Bradbury et al., 2007). The majority of SNPs were found to be in complete disequilibrium, and no LD decay was observed until r² dropped below 0.2 (**Supplementary Figure S2A**). To further investigate the genetic basis of SNP variation, GWAS was performed, and Manhattan plots were generated to visualize significant SNPs exceeding the threshold of −log10(p) > 40 (**Supplementary Figure S2B**). Subsequently, a list of genes containing these significant SNPs was compiled, followed by annotation and GO analysis of the associated gene set (**Supplementary Table S5**). The SNPs with the strongest association on chromosome 1 were linked to MGG02124, which encodes a K+ transporter involved in inorganic ion transmembrane transport (GO:0098660). Additional genes identified included the ammonium transporters MEP1 (MGG00595, MGG00537) and an MFS transporter (MGG00416) on chromosome 5; the secreted chitin deacylase MoCDA1 (MGG14966) on chromosome 6; and MGG02986 on chromosome 7, encoding the DNA polymerase zeta catalytic subunit (REV3), which is essential for DNA replication and repair, particularly in regions of damaged or repetitive DNA. Other notable genes included effector-encoding genes such as MGG_01753, associated with the epigenetic regulator, *MoSET1*.

### 3.4 Genomic landscape of differentiation and genetic diversity

**Figure 3:**
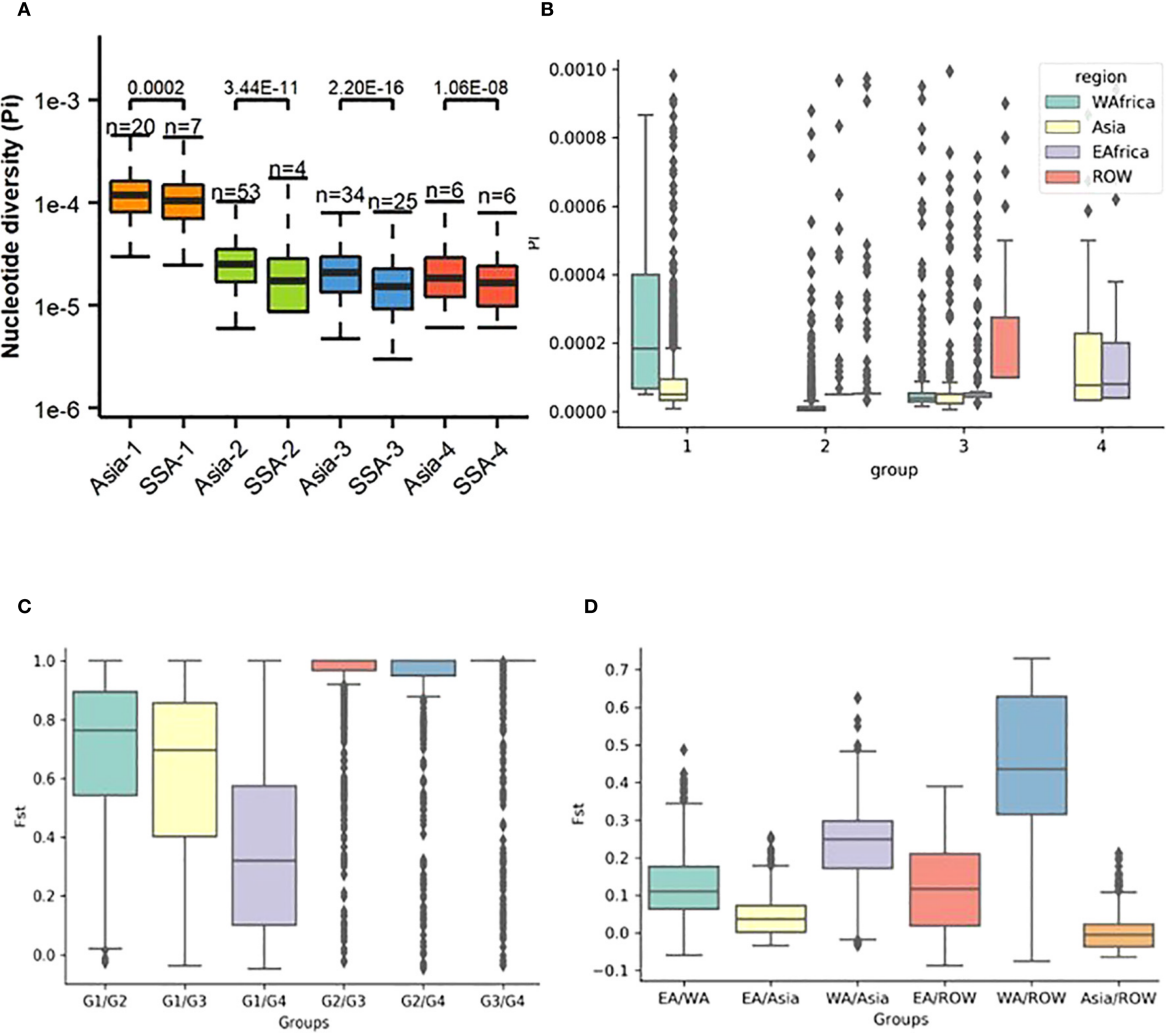
Patterns of diversity across rice-infecting genetic groups in Sub-Saharan Africa (SSA) and Asia. **(A)** Nucleotide diversity (*Pi*) was calculated for each group. **(B)** Nucleotide diversity (*Pi*) compared at the regional level. **(C)** Fixation index (Fst) between different *Pyricularia oryzae* genetic groups across different regions. **(D)** Fst between different regions.

Nucleotide diversity (Pi), fixation index (Fst), and Tajima’s D are widely used in population genetics to characterize population structure and evolutionary dynamics. In our genome-wide analysis, average Pi in Group 1 (Pi = 1.3e-04) was higher than that observed in Groups 2 (Pi = 2.5e-05), 3 (Pi = 2.1e-05), and 4 (Pi = 2.45e-05) (**Figure 3A**; **Supplementary Table S6**). On a regional scale, Pi was observed to display a heterogeneous distribution, with high nucleotide diversity interspersed with stretches of low diversity (**Figure 3B**). Higher Pi was observed in WA Group 1 compared to all other regions and groups. In all regions, lower Pi was observed for Groups 2 and 3.

Low fixation index (Fst) values were observed both across regions (Fst = 0.05) and within each genetic group (Fst = 0.03–0.08) (**Figure 3C**). In contrast, significantly higher Fst values were detected between genetic groups (Fst = 0.25–0.72). Group 1 was found to be highly differentiated from the other groups, with the highest Fst values observed between Groups 1, 2, and 3, while the lowest Fst was recorded between Groups 1 and 4. On a regional scale, significant genetic differentiation was found between WA and Asia, as well as between WA and ROW, with Fst values of 0.22 and 0.42, respectively (**Figure 3D**). Moderate Fst values were observed between EA and Asia and between EA and ROW (both 0.12), whereas the Fst between Asia and ROW was low (0.04).

**Figure 4:**
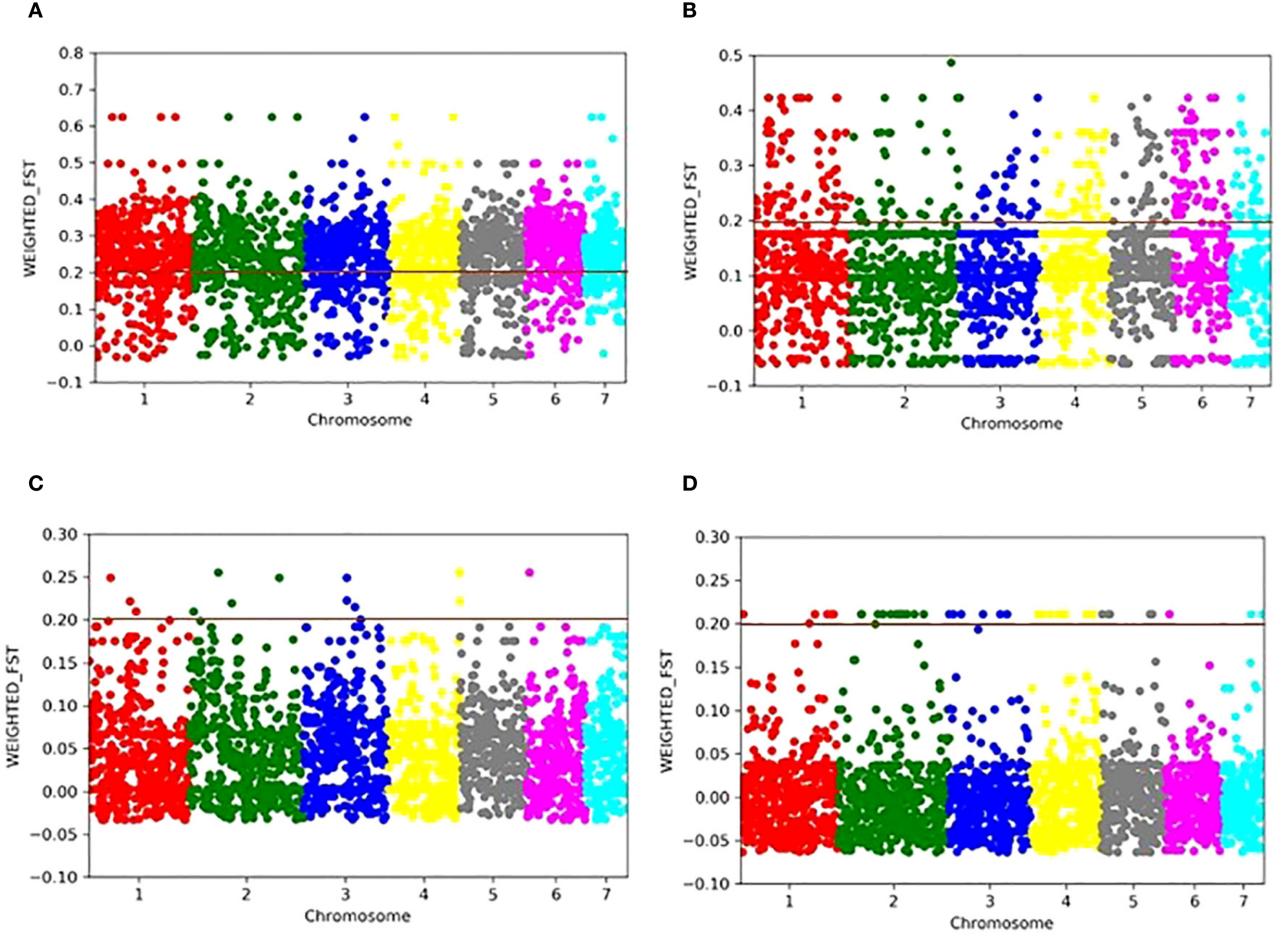
Manhattan plots representing the distribution of pairwise Fst values calculated for each 10 kb genomic window, with an Fst value assigned to each SNP and comparing the distribution of genetic differentiation across the genome between regional populations. **(A)** WA vs Asia, **(B)** WA vs EA, **(C)** EA vs Asia, and **(D)** Asia vs RoW.

To further understand signatures of population differentiation, pairwise Fst values for all individual SNPs were calculated using a 10% SNP threshold across the regions (Asia, EA, WA, and ROW). Outlier SNPs were identified in Manhattan plots, using a weighted Fst threshold of 0.2. Markedly elevated Fst values were observed between WA and Asia, with 1,814 SNPs exceeding the 0.2 threshold (**Figure 4A**). In comparison, only 368 SNPs exceeded this threshold between EA and WA (**Figure 4B**). The differentiation among Asia, EA, and ROW was minimal, with fewer than 30 SNPs above the 0.2 threshold (**Figures 4C, D**). Common SNPs from the highest Fst bins across all chromosomes were pooled to compile a list of linked genes, followed by annotation and GO analysis of the associated gene set. Thirty-eight candidate genes were identified as common between Asia-EA and Asia-WA comparisons (**Supplementary Table S7**) and were linked to diverse functional categories, including ion transport, fungal hyphal growth, conidiation, lipid metabolism, ubiquitination, and fertility. GO term enrichment revealed biological processes such as iron-sulfur cluster assembly (GO:0016226), cellular response to starvation (GO:0009267), cellular response to nutrient levels (GO:0031669), proteasome-mediated ubiquitin-dependent gene expression (GO:0043161, GO:0010467), ncRNA metabolic process (GO:0034660), protein maturation by [4Fe-4S] cluster (GO:0106035), ncRNA processing (GO:0034470), and RNA modification (GO:0009451).

**Figure 5:**
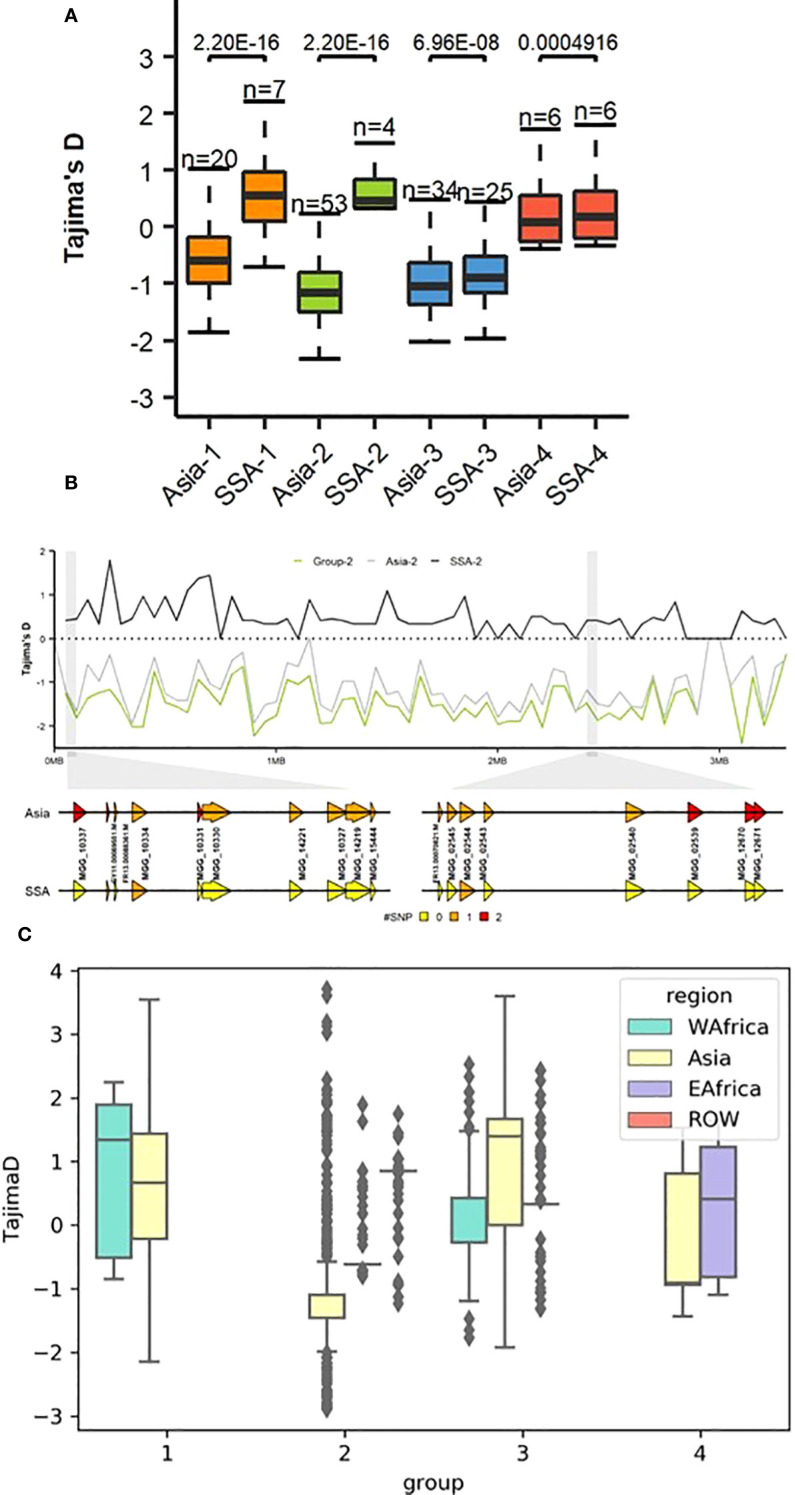
**(A)** Genome-wide Tajima’s *D* computations for genetic groups from SSA and Asia showing substantial differences within groups 1 and 2. To compare the distribution in each genome-wide diversity test analysis for each genetic group within different regions, Mann–Whitney test was performed as shown above in the boxplot. **(B)** Example of the dramatic change in Tajima’s *D* values on *Pyricularia oryzae* chromosome 7 within genetic group 2 from SSA and Asia. Sequence variation of *P. oryzae* genes within a 5 kb window representing color-coded single nucleotide polymorphisms (SNPs). **(C)** Tajima’s D between between regions across genetic groups.

Estimates of Tajima’s D helped to detect deviations from neutrality. Positive Tajima’s D were observed in SSA-1 and SSA-2, but were predominantly around zero (**Figure 5A**). In SSA-4, Tajima’s D was closer to neutrality, consistent with neutral expectations. In contrast, SSA-3 exhibited negative Tajima’s D values, similar to those observed in Asian genetic groups 1, 2, 3 (Asia-1, Asia-2, and Asia-3, Tajima’s D = −0.6 to −1.2), except for Asian genetic group (Asia-4), which showed a value close to zero (**Supplementary Table S8**). Sliding window analysis revealed minor deviations from neutrality across the genome, with more pronounced deviations observed on chromosomes 4, 5, and 7 (**Supplementary Figure S3**), with specific loci potentially involved in adaptation or evolutionary processes in Group 2, highlighted on chromosome 7 (**Figure 5B**). Region-specific analyses revealed that Group 1 in WA exhibited a positive Tajima’s D relative to other regions, with a median greater than 1 (**Figure 5C**). Group 2 in EA also showed high Tajima’s D, although most SNPs fell within the negative range. In Asia-3, Tajima’s D was variable, with most values falling between 0 and 2, while values for West Africa (WA) and EA hovered around 0. In Group 4, Tajima’s D for EA was marginally positive, while in Asia it was negative, with most values near zero. Comparisons for Group 4 did not include WA and ROW due to insufficient numbers of assigned isolates, which limited the ability to conduct reliable analyses.

### 3.5 Population genetic analyses of demographic history

The impact of demographic history was modeled using two types of analyses available in ∂a∂i. Initially, the demographic history of each population was analyzed separately by fitting the default single-population models: expansion (two-epoch function), growth, bottleneck, and bottleneck-expansion (three-epoch function), using an optimal projection size for the site frequency spectra (SFS). However, none of these models were found to adequately fit the data for any of the geographic regions analyzed. Subsequently, two-dimensional demographic models, specifically the split-migration and isolation-migration models, were examined. Among these, the Isolation with Migration (IM) model was identified as providing the best fit for the data and exhibited the highest likelihood values among all paired population comparisons (**Table 1**). In each pairwise comparison, SSA populations emerged from a small proportion of the ancestral population. However, the founding population size of EA was smaller than that of WA. The migration rate from Asia to EA was higher (m12 = 7.29E-03, while m21 = 1.19E-03) than that from Asia to WA (m12 = 1.75E-02, while m21 = 1.70E-02). Analysis of migration between EA and WA yielded no results with the IM model; therefore, the split-migration model was used, which provided a good fit. The joint site frequency spectrum observed for EA-WA indicated an asymmetric split between the two regions (**Supplementary Figure S4**), consistent with the respective founding population sizes. EA and WA had a higher migration rate (m = 0.01) compared to the Asia-EA and Asia-WA population pairs.

**Table 1:** Maximum likelihood parameter estimates for the isolation with migration and split-migration models, for each population pair analyzed.

Dataset	Model	Theta	Likelihood	*S*	Best-fit parameters						
					*N1*	*Nu1*	*Nu2*	*T*	*M*	*M12*	*M21*
Asia-EA	Isolation-migration	3653.92	−10326.76	1.698	0.010	1.700	0.544	0.0070	0.0084	0.007	0.0012
Asia-WA	Isolation-migration	4577.48	−10926.76	1.180	1.034	1.180	1.883	0.0052	0.0066	0.018	0.0170
EA-WA	Split-migration	4218.07	−8745.71	–	0.014	0.010	0.002	0.0084	0.0102	-	-

*S*, population size of the first population after split (population 2 has size 1-s); *nu1*, current size of population 1; *nu2*, current size of population 2; *T*, time of population split; *m*, migration rate; *m12*, migration from population 2 to population 1; *m21*, migration from population 1 to population 2.

### 3.6 Effector distribution and diversification in SSA

**Figure 6:**
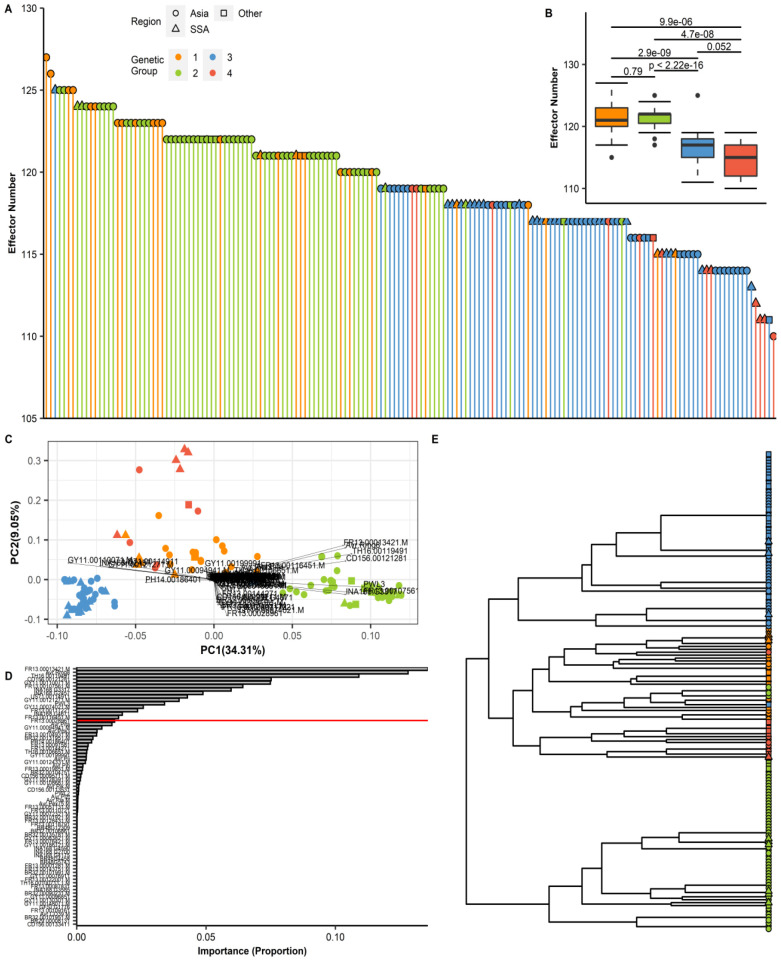
Effector repertoires in *P. oryzae* reveal distinct patterns of diversification in each genetic group. **(A)** An assortment of the total number of effectors per isolate from the highest (CH0333 = 127) to the lowest (IN0072 = 110). **(B)** A box plot of the total effector content in each genetic group. To compare the distribution of the total effector for every isolate in each of the four genetic groups, Mann–Whitney test was performed as shown above for the boxplot. **(C)** Principal component analysis (PCA) biplot of effectors from the presence/absence matrix (**Supplementary Table S9**). The effector loading vectors are indicated by the arrowheads. **(D)** A bar plot showing the product for each effector loading vectors. The redline reveals 90% of the cumulative sum from the data, or in this case, 16 effectors that can explain the distribution. **(E)** Complete hierarchical clustering dendrogram of the 16 effectors based on the results presented in **Supplementary Figure S6E**. The distance matrix was computed using the Jaccard index.

The number and distribution of effector repertoires among different groups and regions were compared by mapping 178 predicted effector references to the genome sequences of *P. oryzae* isolates. The total number of effectors per isolate was found to range from 110 to 127. The highest count was observed in isolate CH0333 (127 effectors), whereas the lowest was detected in IN0072 (110 effectors), with SSA isolates falling within this range. The total effector content across the various genetic groups of *P. oryzae* is presented in **Figure 6A**. When the genetic groups were ranked by effector count, Group 1 was found to possess the largest number, followed by Group 2, while Group 4 displayed the lowest count (**Figure 6B**). Variance in effector presence/absence was visualized using a PCA biplot, which illustrated the contribution of individual effectors to the principal components and their correlations (**Figure 6C**). We observed that some effectors exerted a stronger influence on the principal components, and through effector loading analysis, 16 informative effectors were identified as accounting for 90% (red line) of the cumulative distribution (**Figure 6D**). Visualization of these effectors in a dendrogram revealed two main clusters, with Group 2 forming a distinct cluster separate from Groups 1, 3, and 4 (**Figure 6E**). The presence/absence polymorphism of effectors was evaluated across all genomes, and similar, though not identical, patterns of effector repertoires were observed within each genetic group (**Supplementary Table S9**).

**Figure 7:**
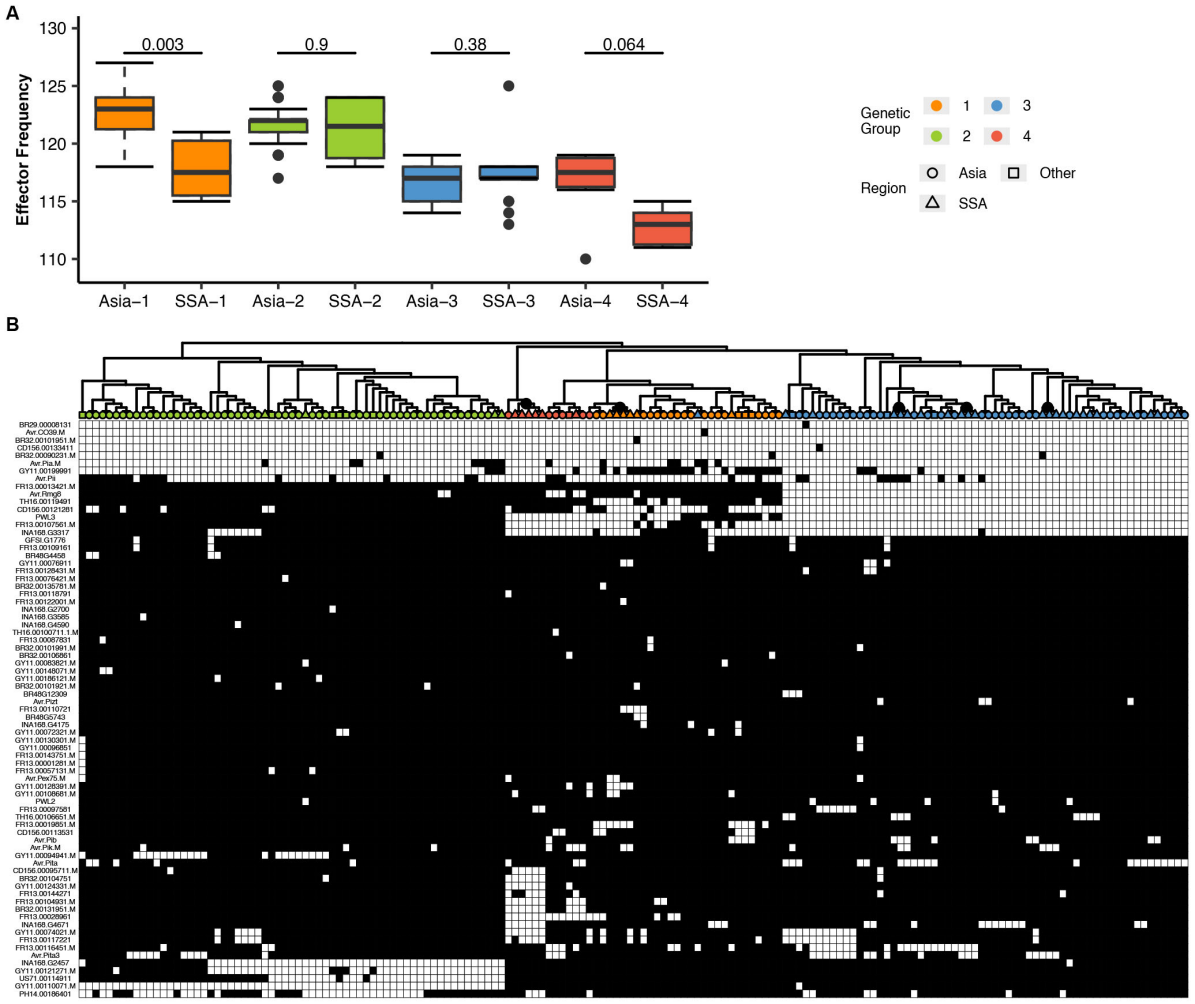
Variation in the number and distribution of candidate effector repertoires in *P. oryzae* genetic groups collected from Sub-Saharan Africa (SSA). **(A)** A box plot representing the average number of effector genes in each genetic group from SSA and Asia. To compare the distribution of each effector content within the genetic group between different regions, Mann–Whitney test was performed as shown above for the boxplot. SSA1–4 and Asia1–4 represent the genetic groups described in **Figure 1**. **(B)** A hierarchical heatmap representing the presence/absence patterns of candidate effectors. SSA (triangles), Asia (circles), and RoW (squares) regions are depicted. Color labels in the tree represent the genetic group. Gray and white colors in the heat map represent the presence/absence of effectors as <80% of coverage. Gene names (rows) and isolate names (columns) are described in **Supplementary Table S9**. Subclusters in genetic groups 1, 3, and 4 are indicated as gray nodes in the tree. Complete-linkage clustering was performed as visualized in the dendrogram.

To assess genetic diversity of *P. oryzae* effectors across regions, consensus sequences for all effector genes were extracted and variants identified. Overall diversity, measured as the probability of encountering different effector gene variants, was higher in Asia than in SSA across all genetic groups (**Figure 7A**). Within genetic groups 1, 3, and 4, distinct subclusters of effector patterns from SSA *P. oryzae* genomes were observed (**Figure 7B**). Sequence variation among candidate effector repertoires was analyzed to detect variation in genetic groups for SSA and Asia genomes. Variation in effector genes was significantly higher in Asia than in SSA, as shown in the box plot (**Supplementary Figure S5A**). A similar trend was observed for effector nucleotide diversity (**Supplementary Figure S5B**). Likelihood ratio tests showed that dN/dS ratios were largely comparable among genetic groups, with the highest ratio observed in Group 2 (**Supplementary Figure S5C**). Differences in the allelic frequencies of effectors for each genetic group were visualized using a heatmap (**Supplementary Figure S6A**), and this confirmed higher allele frequencies in Asia compared to SSA, regardless of genetic group. The number of effector genes with sites under positive selection, as predicted by CODEML, increased to 36; however, only six effectors exhibited multiple sites with dN/dS > 1 (**Supplementary Figure S6B**). These included MGDIG41 (detected in all isolates), Avr.Pita3 (with presence/absence polymorphism), FR13.0004761 (detected in all isolates), FR13.00128431 (with presence/absence polymorphism), NA168 (detected in all isolates), and AvrPex75 (with presence/absence polymorphism). Two effectors, INA168.G2457 and FR13.00128431, exhibited a wide range of dN/dS values from 0 up to 99, followed by FR13.00094761 and MGDIG41, which showed sparse dN/dS values within the same range (**Supplementary Figure S6C**). Regional differences in positive selection signatures occurred predominantly in Group 1, with WA isolates displaying a higher number of positively selected sites compared to those from Asia and EA (**Supplementary Figure S6D**).

## 4 Discussion

Population analyses is crucial for understanding the evolutionary histories and forces shaping pathogen populations, which in turn inform management strategies. The resolution and accuracy of genetic diversity estimates and other population genetic inferences have been greatly improved by the use of genomic markers, allowing a finer dissection of pathogen population structure, ancestry, and adaptive evolution.

In this study, we analyzed 180 genome sequences of *P. oryzae*, including 45 newly sequenced isolates from SSA, to uncover patterns of genetic variation, population structure, demographic history and effector gene diversity. Our analyses revealed that SSA populations have diverse ancestries, consistent with multiple introduction events, a finding that corroborates previous studies (Thierry et al., 2022). However, isolates from WA exhibited considerable genetic divergence from Asian populations compared to other regions, suggesting region-specific evolutionary trajectories. Phylogeographic reconstruction placed the most recent common ancestor (MRCA) of all isolates in this study around 1742 CE, considerably more recent than the global evolutionary timeline of *P. oryzae*, which extends over 12,000 years (Couch et al., 2005; Zhong et al., 2017; Latorre et al., 2020). To reconstruct the pathways of pathogen migration, we employed spatial simulations and Bayes factor analyses of diffusion rates, supported by visualization tools such as Google Earth. Our results revealed two primary migration waves from China to SSA: (1) a direct introduction, and (2) a secondary, indirect route involving intermediary countries. Most SSA introductions were traced directly to China, most probably associated with the significance of historical agricultural projects in facilitating pathogen dispersal, a phenomenon also documented for other plant pathogens (Brasier, 2008). A compelling case is provided by the genetic grouping of Chinese and Ugandan isolates, notably E-UGD-32 from the TILDA irrigation scheme, which was established with Chinese support in the 1970s. This isolate clustered closely with Chinese isolates 13FM-5–1 and 13FM-24–1 from Yunnan province. Group 1 isolates from WA countries (e.g., Mali, Togo, Burkina Faso, Nigeria) also had Asian ancestry genetic signatures, yet with considerable divergence from contemporary Asian genomes. This suggests that after their introduction, these populations have evolved independently, likely influenced by region-specific host-pathogen interactions, adaptation to local rice varieties, and demographic events.

The second, more recent migration wave is characterized by *P. oryzae* isolates that were initially introduced to other countries, such as South Korea, the Philippines, and India, from China, and subsequently introduced into SSA. The genetic connection between Mali isolates and both China and South Korea, for instance, highlights the complexity of such pathogen movements. These multidirectional introductions are plausibly linked to periods of intensified human movement and trade, especially during and after the European colonization of Africa. Colonial expansion not only transformed agricultural practices but also led to the widespread importation of Asian rice varieties (Reid, 2002; Vaughan et al., 2008; Uma, 2022). Historical records show that rice became increasingly important as a staple in several African countries during the colonial era, which is in line with our phylogeographic inferences regarding the timing of *P. oryzae* emergence in the region (Small, 1922). This pattern mirrors the introduction of other significant plant pathogens, such as *Phytophthora infestans* in the Cape Peninsula, a key node in the Europe-India trade route (Blersch, 1890; Goss et al., 2014). Together, these findings underscore how geopolitical and socio-economic transformations inadvertently created opportunities for pathogen migration and establishment in previously uninfected regions (Stukenbrock and McDonald, 2008). Within SSA, continued *P. oryzae* spread among countries appears to be driven by the movement of planting materials and regional trade (Mutiga et al., 2021). Notably, we observed significant transmission events between Burkina Faso and Burundi, as well as connections between Côte d’Ivoire, Nigeria, and Ghana with Burundi. Burundi’s central position in the Great Lakes region may have contributed to its role as a hub, while Madagascar’s relative isolation likely limited international connections. Additional connections, such as those between Ghana and Benin, and between Kenya and Uganda, with both East African countries also showing links to Tanzania, further underscore the networked nature of pathogen movement within SSA, potentially aligned with trans-African transport corridors (FAO, 2019).

Population structure analyses revealed that Groups 3 and 4 in SSA are more closely related to Group 1 than to Group 2, supporting previously proposed divergence patterns (Gladieux et al., 2018b; Latorre et al., 2020). Group 3, comprising about two-thirds of the regional population, displayed a wide geographic spread and a coherent structure in STRUCTURE analysis, a pattern indicative of successful regional expansion and relative clonal stability. Such widespread distribution of a clonal lineage is a hallmark of epidemic populations of plant pathogens, where a few highly adapted genotypes proliferate across large areas, often due to their fitness advantages in prevailing agroecological and host environments (McDonald and Linde, 2002; Saleh et al., 2014). In contrast, WA Group 1 isolates showed increasing separation from Asian groups at higher K values. This divergence suggests that, following their initial introduction, WA Group 1 isolates have undergone local evolution, possibly shaped by adaptation to local rice varieties and reduced recent gene flow from Asia (Latorre et al., 2020; Gladieux et al., 2018b). Conversely, Group 1 isolates from EA consistently clustered with Asian populations even at higher K values, indicating more recent transmission event from Asia into EA or less disrupted genetic diversity compared to WA. These patterns are further supported by f_2_ statistics, which revealed weak genetic connectivity between EA and WA in Group 1 and a strong relationship between the two regions in Group 3. The consensus f2 admixture tree placed EA Group 1 isolates at the base of a lineage leading to Group 2, indicating that their ancestral lineage likely served as the progenitor for Group 2 isolates. In contrast, WA Group 1 isolates were positioned at the base of Group 4, which traces back to the broader Group 1 lineage leading to group 3. This divergence occurred considerably later than the split of EA Group 1 from the broader Asian Group 1. Group 3 was inferred to have descended from Southeast Asian Group 1. Prior to the emergence of SSA-3 (EA and WA) cluster, two distinct Asian subpopulations, corresponding to South and East Asia, were evident, suggesting that SSA-3 originated from a Southeast Asian lineage that had already diverged into these subregions. This phylogenetic pattern provides additional evidence that independent introductions and subsequent local evolution, rather than a single pan-continental dispersal event, have shaped the population structure of *P. oryzae* in SSA. This is consistent with previous studies showing that multiple, temporally and spatially separated pathogen introduction events, often driven by human migration, long-distance trade, and shifting agricultural practices, contribute to local adaptation and diversification (Gladieux et al., 2018b; Latorre et al., 2020).

Demographic modeling using *dadi* suggested that both WA and EA descended from small founding populations, consistent with the hypothesis that *P. oryzae* populations outside Asia originated from a limited number of founders (Levy et al., 1991; Zeigler, 1998). Such founder events can significantly reduce genetic diversity and increase the influence of genetic drift, shaping the initial genetic landscape of introduced populations (McDonald and Linde, 2002). These founder effects may have played a crucial role in shaping the genetic profiles, particularly in WA, compared to those of their ancestral populations. Divergence time estimates indicate a more recent separation between WA and Asia, and greater divergence in WA. This greater differentiation in WA likely reflects not only the historical impact of founder events but also ongoing processes of local adaptation, which may have been driven by the region’s unique host diversity following the introduction and intensification of rice cultivation.

Genetic diversity metrics further support these conclusions: WA isolates, especially those in Group 1, showed higher *Pi* and positive Tajima’s D compared to their Asian and EA counterparts. Elevated Pi suggests a broader spectrum of genetic variation within WA populations, while positive Tajima’s D is indicative of the maintenance of multiple alleles at intermediate frequencies. Such patterns can arise from balancing selection, where diverse host-pathogen interactions favor the persistence of different alleles, or from the retention of intermediate-frequency variants following historical population bottlenecks (Tajima, 1989; Charlesworth, 2006). In plant pathogen systems, balancing selection is often driven by the coexistence of varied host genotypes and fluctuating selection pressures imposed by the deployment of resistance genes (Stukenbrock and McDonald, 2008). These genetic diversity patterns are further corroborated by genome-wide pairwise F_ST_ analyses and cross-population scans, which revealed pronounced genetic differentiation between WA and Asian populations. Specifically, a higher number of outlier SNPs and candidate genes potentially linked to adaptation were identified in WA isolates. Previous studies have shown that such differentiation can be driven by both geographic barriers and the distinct evolutionary trajectories imposed by the independent domestication and diversification of rice in WA (Mutiga et al., 2021). Consistent with these findings, our analysis also revealed a higher number of positively selected effector sites in WA isolates than in other regions, further supporting the hypothesis that diversification of rice cultivars in WA has driven local adaptation of *P. oryzae*. This is consistent with broader findings that regions with greater host diversity tend to harbor pathogens with more diverse and rapidly evolving effector repertoires (Stukenbrock and McDonald, 2008). The domestication of *Oryza glaberrima* in WA approximately 3200 BP likely provided a long-standing, genetically distinct host environment, which was later complemented by the introduction of Asian *O. sativa* (Linares, 2002). The coexistence and interaction of these two rice species likely created selection pressures, favoring localized adaptation and increased genetic divergence in WA *P. oryzae* populations. Similar patterns of pathogen adaptation following host diversification have been observed in other agroecosystems, where the interplay between historical introductions, host genetic resources, and local selection has driven both population structure and evolutionary trajectories (Stukenbrock and McDonald, 2008; Croll and Laine, 2016).

The relatively modest genetic differentiation observed between WA and EA, as evidenced by lower F_ST_ values and fewer genomic outlier regions, suggests more recent or ongoing gene flow and/or the retention of shared ancestral alleles between these regional populations. Such genetic connectivity is frequently maintained through the regular exchange of rice germplasm and seeds, as well as similar agroecological conditions and farming practices across neighboring countries (Gladieux et al., 2018b; Thierry et al., 2022). Previous studies have demonstrated that the movement of planting materials, often facilitated by regional trade and agricultural development programs, plays a central role in shaping the genetic structure and connectivity of plant pathogen populations in Africa (Mutiga et al., 2021). Consistent with this interpretation, both WA and EA populations showed near-zero Tajima’s D values in clonal Groups 3 and 4, which is indicative of either weak balancing selection, where multiple alleles are maintained within the population or demographic neutrality, where neither strong expansion nor contraction dominates the population’s evolutionary trajectory (Charlesworth, 2006). Such patterns are often observed in more established populations or where there is ongoing gene flow and relatively stable host-pathogen dynamics (Stukenbrock and McDonald, 2008). This observed genetic similarity and neutrality in Tajima’s D in SSA Groups 3 and 4 may, in part, be attributed to the widespread adoption of New Rice for Africa (NERICA) varieties, which are interspecific hybrids containing alleles from both *O. glaberrima* and *O. sativa*. The expansion of NERICA and similar varieties across both WA and EA has likely contributed to the convergence of host environments, imposing similar selection pressures on *P. oryzae* populations and resulting in parallel patterns of genetic diversity and demographic stability.

Furthermore, most effector genes analyzed in this study exhibited patterns consistent with purifying selection or functional conservation, particularly within Groups 3 and 4. This suggests that, despite geographic separation, the evolutionary forces acting on effector repertoires are comparable in both WA and EA. Such patterns align with previous findings that, while a subset of *P. oryzae* effector genes evolves rapidly, presumably to escape recognition by host immune systems, the majority remain highly conserved due to their critical roles in pathogen virulence (Xue et al., 2012; Kim et al., 2019). An exception to this trend was observed in Group 2, where marked divergence in effector repertoires was found relative to other groups. Similar patterns of group-specific effector content have previously been reported in *P. oryzae* lineages, highlighting the importance of lineage-specific adaptation (Dong et al., 2015). We also found notable differences in effector gene presence/absence between Asia-1 and SSA-1, and the emergence of SSA specific subclusters within SSA-3. These SSA-specific effector alleles may represent ancient variants retained in African populations but lost in Asia, or recent adaptations to the unique host diversity and agroecological conditions of SSA rice-growing systems. This contrasting effector distributions between Asian and SSA populations underscore the impact of local selection pressures and historical contingency on the effectorome structure of *P. oryzae*. MAX effectors, a prominent and highly variable effector family in *P. oryzae*, are known to undergo rapid sequence evolution, as evidenced by increased rates of non-synonymous substitutions under diversifying selection (Le Naour—Vernet et al., 2023). Our analyses found only a subset of MAX effectors that displayed signatures of positive selection, such as INA168.G2457 and FR13.00128431, which had highly variable dN/dS ratios. Collectively, these results highlight the dual forces of purifying selection and localized positive selection acting on the *P. oryzae* effectorome in SSA. This interplay drives both the maintenance of essential virulence functions and the rapid adaptation to the changing host populations, underscoring the importance of integrating effector diversity analyses into pathogen surveillance and management strategies.

Our study is not without limitations. The sampling strategy focused on epidemic hotspots and available archival samples, potentially leading to underrepresentation of certain regions and introducing biases in estimates of diversity and structure. Additionally, many population genetic methods assume random mating and sexual reproduction, while *P. oryzae* is predominantly clonal, which may bias estimations of linkage disequilibrium and effective population size. Although we have taken steps to minimize such biases by using genome-wide markers and robust analytical approaches, interpretations of our results, particularly, population structure and demographic history should be made with caution.

In conclusion, our findings reveal a dynamic landscape of *P. oryzae* evolution in SSA, shaped by historical introductions and local adaptation. While WA and Asian populations of *P. oryzae* have experienced significant divergence, populations within SSA (WA and EA) remain more interconnected, underscoring the importance of regional migration and common ancestry in shaping present-day diversity. Patterns of effector gene diversity and selection were identified, providing insights into historical contingency on the effectorome structure of *P. oryzae* in SSA and Asia. Collectively, these results highlight the need for integrated strategies that account for both historical and contemporary pathogen dynamics when designing biosecurity protocols and disease management programs in Africa.

## Data Availability

The sequences produced in this study are stored under BioProject PRJNA670311.

## Supplementary material

Supplementary Figure 1 Four genetic groups inferred in global *Pyricularia oryzae* isolates based on the whole genome single nucleotide polymorphisms. **(A)** Bar plot showing the membership probability for each genome from K = 2 to K = 8 populations. The clusters were built using a discriminant analysis of principal components (DAPC). **(B)** The Bayesian information criterion (BIC), and **(C)** the Silhouette score hinting at four genetic groups as the optimum number of clusters (elbow in the BIC curve and maximum Silhouette score).

Supplementary Figure 2
**(A)** Linkage disequilibrium (LD) estimated using 66,744 single nucleotide polymorphisms (SNPs). Decay of r^2^ as a function of genetic distance between SNP markers. **(B)** Genome-wide Manhattan plot for seven *Pyricularia oryzae* chromosomes showing the results of genome wide association analysis and the significant SNPs linked to candidate genes.

Supplementary Figure 3 Genome-wide Tajima’s *D* for *Pyricularia oryzae* chromosomes revealing different patterns in genetic group 1 and 2 across regions. Tajima’s *D* for each genetic group from chromosomes 1 to 7 was built using a 50 kb sliding window. The color for the different genetic groups corresponds to the predicted groups in **Figure 1** and **Supplementary Figure S2**. Tajima’s *D* values for Asia and Sub-Saharan Africa (SSA) regions are depicted as grey and black lines.

Supplementary Figure 4 The observed joint site frequency spectrum for *Pyricularia oryzae* in, **(A)** Asia-East Africa (Asia-EAfrica), **(B)** Asia-West Africa (Asia-WAfrica), and **(C)** East Africa-West Africa (EAfrica-WAfrica) along with the model fit and residuals. Residuals for each model are plotted below their expected spectra.

Supplementary Figure 5 Diversity of *Pyricularia oryzae* effectors associates with a strong purifying selection. **(A)** Allele diversity analysis of each effector from the four genetic groups. **(B)** Nucleotide diversity (*Pi*) of each effector from the four genetic groups. **(C)** Ka/Ks effector distribution in each genetic group. To compare the distribution of each effector diversity test analysis in each of the four genetic groups, Mann–Whitney test was performed.

Supplementary Figure 6
**(A)** Allele diversity of effector repertoires in *Pyricularia oryzae* genetic groups from Asia and Sub-Saharan Africa (SSA). The heatmap shows allele frequency from effectors present in each genetic group across regions. Effector alleles range from 1 to 16. More effector alleles are present in Asia compared with that in SSA, regardless of the genetic group. Low frequency (yellow) and high frequency (red) is based on the total number of isolates with that particular allele in each effector. The color in the text is based on the four genetic groups. **(B)** Boxplot: effectors with most sites under positive selection are shown among the 36 effectors. Region-wise, dn/ds values exhibit considerable diversity in distribution.
